# A genome-wide association study for genetic susceptibility to *Mycobacterium bovis* infection in dairy cattle identifies a susceptibility QTL on chromosome 23

**DOI:** 10.1186/s12711-016-0197-x

**Published:** 2016-03-09

**Authors:** Ian W. Richardson, Donagh P. Berry, Heather L. Wiencko, Isabella M. Higgins, Simon J. More, Jennifer McClure, David J. Lynn, Daniel G. Bradley

**Affiliations:** Smurfit Institute of Genetics, University of Dublin, Trinity College, Dublin 2, Ireland; Animal and Grassland Research and Innovation Centre, Teagasc, Moorepark, Fermoy, Co. Cork Ireland; Animal and Bioscience Research Department, Animal and Grassland Research and Innovation Centre, Teagasc, Grange, Co. Meath Ireland; UCD Centre for Veterinary Epidemiology and Risk Analysis, UCD School of Veterinary Medicine, University College Dublin, Belfield, Dublin 4, Ireland; Irish Cattle Breeding Federation, Bandon, Co. Cork Ireland; South Australian Health and Medical Research Institute, North Terrace, Adelaide, SA 5000 Australia; School of Medicine, Flinders University, Bedford Park, SA 5042 Australia

## Abstract

**Background:**

Bovine tuberculosis (bTB) infection in cattle is a significant economic concern in many countries, with annual costs to the UK and Irish governments of approximately €190 million and €63 million, respectively, for bTB control. The existence of host additive and non-additive genetic components to bTB susceptibility has been established.

**Methods:**

Two approaches i.e. single-SNP (single nucleotide polymorphism) regression and a Bayesian method were applied to genome-wide association studies (GWAS) using high-density SNP genotypes (n = 597,144 SNPs) from 841 dairy artificial insemination (AI) sires. Deregressed estimated breeding values for bTB susceptibility were used as the quantitative dependent variable. Network analysis was performed using the quantitative trait loci (QTL) that were identified as significant in the single-SNP regression and Bayesian analyses separately. In addition, an identity-by-descent analysis was performed on a subset of the most prolific sires in the dataset that showed contrasting prevalences of bTB infection in daughters.

**Results:**

A significant QTL region was identified on BTA23 (P value >1 × 10^−5^, Bayes factor >10) across all analyses. Sires with the minor allele (minor allele frequency = 0.136) for this QTL on BTA23 had estimated breeding values that conferred a greater susceptibility to bTB infection than those that were homozygous for the major allele. Imputation of the regions that flank this QTL on BTA23 to full sequence indicated that the most significant associations were located within introns of the *FKBP5* gene.

**Conclusions:**

A genomic region on BTA23 that is strongly associated with host susceptibility to bTB infection was identified. This region contained *FKBP5*, a gene involved in the *TNFα*/*NFκ-B* signalling pathway, which is a major biological pathway associated with immune response. Although there is no study that validates this region in the literature, our approach represents one of the most powerful studies for the analysis of bTB susceptibility to date.

**Electronic supplementary material:**

The online version of this article (doi:10.1186/s12711-016-0197-x) contains supplementary material, which is available to authorized users.

## Background

Bovine tuberculosis (bTB; caused by infection with *Mycobacterium bovis*) is an important infectious disease, that affects primarily cattle. It is an OIE (World Organisation for Animal Health) listed disease (https://www.oie.int), with global annual losses to agriculture of €2 billion [1]. The primary cost of bTB infection in developed countries relates to efforts to control the disease, with recent estimates of annual costs of €63 million, €29 million and €190 million to the Irish, Northern Irish [2], and UK governments [2, 3]. Many European countries are currently faced with bTB, but the problem is most acute in Ireland and the UK in spite of national eradication programs since the late 1950s [4].

Both within-breed [5–7] and between-breed [5, 8] variation in susceptibility to bTB in dairy and beef cattle have been reported. Documented heritability estimates for susceptibility to bTB in cattle range from 0.08 to 0.19 [5–7]. Heritability estimates for Johne’s disease (i.e., infection with *Mycobacterium avium* ssp. paratuberculosis), which shares some epidemiological similarities with bTB [9], range from 0.07 to 0.15 [10–12].

Although several genome-wide association studies (GWAS) have been undertaken on Johne’s disease in cattle [13–15], only three have been initiated on bTB [16, 17], i.e. (1) Finlay et al. [16] performed a medium-density GWAS on a population that consisted of the progeny of 307 Holstein–Friesian bulls and suggested that a genomic region on BTA22 (BTA for *Bos taurus* chromosome) was putatively associated with bTB susceptibility [18]; (2) Bermingham et al. [17] carried out a high-density GWAS on a population of 1151 Holstein–Friesian cows (592 cases and 559 age-matched controls) and identified two genes that were putatively associated with bTB susceptibility: *PTPRT* on BTA13 and *MYO3B* on BTA2; however, the SNPs that were identified as significantly associated with bTB susceptibility by Finlay et al. [16] were not validated by Bermingham et al. [17] and vice versa; and (3) Kassahun et al. [19] used an admixture mapping approach with a low-density marker panel on African-European bovine hybrid field populations and provided evidence of an association between bTB susceptibility and a region that carries several *toll*-*like receptor* genes on BTA6.

Several GWAS on susceptibility to human tuberculosis (TB, caused by infection with *M. tuberculosis*) have also been performed on human populations [20]. Several genes were identified and validated, including the genes *NRAMP1*, *IFNG*, *NOS2A*, *MBL*, *VDR* and four *toll*-*like receptor* genes [20]. A whole-genome scan on mice that were experimentally infected with *M. tuberculosis* [21] identified loci in the region of the *sst1* gene that is involved in the immune response of macrophages to TB [20]. Several single gene investigations for bTB-related traits have been reported and *NRAMP1* (*SLC11A*) was identified as a susceptibility locus in both cattle and humans [22–24].

The objective of our study was to detect regions of the bovine genome associated with bTB susceptibility in Irish dairy cattle and elucidate any likely candidate genes and pathways that contribute to these putative quantitative trait loci (QTL). High-density genotypes of dairy bulls (n = 841) used for artificial insemination (AI) were associated with estimated breeding values (EBV) for bTB susceptibility that had been calculated from epidemiological information on 105,914 daughters. This information was used in two distinct GWAS approaches and a haplotype association approach, to search for QTL associated with bTB susceptibility in dairy cattle.

## Methods

### Ethics and consent

Animal Care and Use Committee approval was not obtained for this study because all data were from the pre-existing database infrastructure operated by the Irish Cattle Breeding Federation database (ICBF, Bandon, Co. Cork, Ireland).

### Phenotypic data

Individual animal EBV for bTB susceptibility were calculated using an animal linear mixed model in ASREML [25]. Details on the data, statistical model and variance components used to estimate the EBV are available in Richardson et al. [5]. In brief, we used as dependent trait the results of single intra-dermal comparative tuberculin tests (SICTT) [26, 27] (i.e., it was a binary trait) that were obtained on individuals that were present during one of the 4048 herd bTB episodes (i.e. periods of herd restriction that are triggered by the detection of bTB infection in one or more animals) in 3240 Irish dairy herds. Several data editing criteria, described in detail in Richardson et al. [5], were applied to maximise the likelihood that all animals that were enrolled from each bTB episode had been equally exposed to bTB. After data editing, 105,914 SICTT records on cows remained. These cow records were split across dairy (n = 87,918), beef (n = 10,202) and mixed herds (n = 7794), a total of 4977 EBV for Holstein sires were calculated using these records. Only the EBV of sires with a reliability of at least 0.30 were retained for the GWAS.

Sire EBV from the animal model were deregressed as:

$${\tilde{\mathbf{y}}} = {\mathbf{R}}\left( {{\mathbf{R}}^{ - 1} + {\mathbf{A}}^{ - 1} } \right){\hat{\mathbf{a}}},$$

where ${\tilde{\mathbf{y}}}$ is a vector of deregressed EBV, **R** is a diagonal matrix containing one divided by, one minus the animal’s reliability from its progeny, **A** is the numerator relationship matrix among animals and ${\hat{\mathbf{a}}}$ is a vector of EBV.

### Genotypes

Illumina high-density (HD) SNP genotypes (n = 777,962 SNPs) were available on 770 Holstein–Friesian bulls. All animals had a genotype call rate greater than 95 %. When genotypes were available for both sire and son(s), the proportion of Mendelian inconsistencies between sire-son pairs was determined to confirm true parentage. A total of 3554 autosomal SNPs that displayed more than 2 % Mendelian inconsistencies were discarded. The genotypes of both the sire and son were set to missing for the remaining autosomal SNPs for which sporadic Mendelian inconsistencies existed.

A total of 1574 SNPs with an Illumina GenTrain score less than 0.55, 40,934 non-autosomal SNPs and duplicate SNPs on the Illumina high-density array were also discarded. Of the remaining SNPs, 17,273 with a call rate less than 95 %, 83,833 SNPs with a minor allele frequency less than 0.02 and SNPs that deviated (P < 0.1 × 10^−8^) from the Hardy–Weinberg equilibrium were excluded. After editing, 597,144 autosomal SNPs remained for the analyses. Missing genotypes were imputed using Beagle [28, 29].

Illumina Bovine50 beadchip genotypes (i.e. 54,001 SNPs) were available on 5313 Holstein–Friesian dairy bulls; 639 of these animals also had HD genotypes. Animal and SNP editing criteria were as previously described and 45,239 SNPs remained after editing. Genotypes were imputed to HD for each chromosome separately using BEAGLE [28–30] (BTau 4.6.1 reference build) and the available 639 HD genotypes were used as reference. A total of 579 Holstein–Friesian bulls with HD genotypes and an additional 262 Holstein–Friesian bulls with imputed genotypes had EBV for bTB susceptibility with reliabilities greater than 0.30. These 841 bull SNP genotypes (597,144 SNPs) were used in GWAS for bTB susceptibility.

### Association analyses

Two alternative strategies were used to test for genome-wide association with bTB susceptibility: a single-SNP regression approach and a Bayesian approach. Prior to inclusion in the association analyses, the deregressed EBV were weighted using the formula outlined by Garrick et al. [31]:

$$\omega_{i} = \frac{{1 - h^{2} }}{{\left[ {c + \left( {1 - r_{i}^{2} } \right)/r_{i}^{2} } \right]h^{2} }},$$

where *ω*_*i*_ is the weighting factor of the *i*th EBV, *h*^2^ is the heritability estimate for bTB susceptibility (here, an *h*^2^ of 0.14 was assumed [5]), $r_{i}^{2}$ is the reliability of the *i*th EBV and *c* is the proportion of genetic variance not accounted for by the SNPs. Weighting factors were calculated with a *c* value of 0.10 for the Bayesian approach and 0.90 for the single-SNP regression approach.

### Using the single-SNP regression method in genome-wide analyses

SNP effects for bTB susceptibility were estimated using mixed models implemented in WOMBAT [32, 33]. Weighted deregressed EBV were the dependent variable and individual was included as a random effect with relationships among animals accounted for via the pedigree relationship matrix. Allele dosage for each SNP was included in the model as a fixed effect. Significance levels for each SNP were calculated from the resulting t-statistic assuming a two-tailed t-distribution. SNPs with a P value less than 1 × 10^−5^ were considered to be genome-wide significant based on a false discovery rate of 1 % [34]. The genetic variance attributable to each SNP was calculated as 2*pqa*^2^, where *p* was the major allele frequency, *q* the minor allele frequency and *a* the allele substitution effect.

### Bayesian approach

Estimation of SNP effects for bTB susceptibility using a Bayesian model was implemented in the GenSel software package [35]. Weighted deregressed EBV was the dependent variable. A BayesC model averaging approach as described by Kizilkaya et al. [36] was first implemented to estimate the genetic and residual variances for bTB susceptibility. BayesC assumes a common variance for all SNPs in the model [35]. Starting values for the genetic and residual variances for BayesC were estimated using a linear mixed model implemented in ASREML [25].

A BayesB algorithm, as described by Meuwissen et al. [37], was subsequently used to estimate SNP effects. The genetic and residual variances used for bTB susceptibility were those that were estimated with the BayesC algorithm. The posterior probability that a SNP was not associated with susceptibility to bTB infection (π) was assumed to be equal to 0.999. A burn-in of 10,000 and chain length of 41,000 were applied in both BayesC and BayesB algorithms. Bayes factors (BF) for each SNP were calculated from the BayesB analysis as an alternative to classical frequentist hypothesis testing. These were calculated as [38]:

$$BF_{i} = \left( {f_{i} /(1 - f_{i} ))/(\pi_{1} /\pi } \right),$$

where *f*_*i*_ is the number of times SNP *i* was included in the model, *π* is the posterior probability that SNP *i* is not associated with bTB susceptibility, and *π*_1_ is the posterior probability that SNP *i* is associated with bTB susceptibility (represented as 1 − *π*). Bayes factors greater than 3.2, 10 and 100 are considered as ‘substantially’, ‘strongly’ and ‘decisively’ associated with a trait, respectively [39]. The proportion of genetic variance accounted for by each SNP was calculated from the estimated effect and allele frequency using GenSel.

### Defining QTL regions

QTL regions of interest were defined based on linkage disequilibrium (LD) around each SNP that had a genome-wide association significance level of P < 1 × 10^−5^ or a Bayes factor greater than 10. Pairwise LD, as measured by r^2^ between all SNPs within 5 Mb upstream and downstream of significant SNPs, was calculated using PLINK [40]. Within these 10-Mb regions, r^2^ was set to 0.5 or more and the SNPs that were furthest upstream and downstream of a significant SNP with this level of LD were used to indicate the beginning and end of a QTL. QTL that overlapped with one another were combined into a single QTL. These QTL were then investigated for the presence of genes within these regions.

### Network analysis

Genes that are present in QTL regions can be used to construct a biological network from available biological network databases, such as InnateDB [41]. Such networks can then be investigated to identify over-represented pathways/ontologies in the network or sub-networks. Network analysis was performed separately on the gene lists that were derived from the single-SNP regression and the Bayesian analyses. Genes that were identified within the QTL regions were sent as a query to the InnateDB website [41] to obtain lists of interacting networks that were over-represented in any pathway that may be associated with bTB susceptibility. The analysis was performed using the hypergeometric algorithm and the Benjamini Hochberg correction for multiple hypotheses testing.

### Identity-by-descent analysis

A subset of prolific AI sires (i.e. with more than 50 daughters in more than 10 herds) that had been identified by Richardson et al. [5] as having a mean daughter prevalence of bTB infection greater than 60 % (n = 11) was used as a case group to investigate the presence of identical-by-descent (IBD) haplotypes that may be associated with bTB susceptibility. The control group was a subset of the most prolific AI sires that had been identified by Richardson et al. [5] and had a mean daughter prevalence of bTB infection less than 30 % (n = 154). Genotypes of the 11 cases and 154 controls were phased using BEAGLE [28, 29] and pair-wise IBD segments were called using fastIBD, an algorithm implemented in BEAGLE. The proportion of genome IBD shared within case and control groups was calculated by dividing the total amount of genome IBD shared per segment by the total number of possible pairs in the case or control groups (i.e., n*(*n* − 1)/2*n*), where *n* is the number of animals within each group. The proportions of genome IBD per segment for the case group were normalized against the proportions of genome IBD per locus for the control group as follows:

$$\frac{{(X - Y)^{2} }}{2X},$$

where *X* is the case–case IBD proportions and *Y* is the control–control IBD proportion, per segment.

### Targeted imputation of sequence data in candidate regions

3-Mb sequence windows that flanked the significant QTL regions (P < 1 × 10^−5^, Bayes factor >10 and normalized proportion of genome IBD >0.5) across all three analyses were imputed from HD to full sequence. Imputation was performed with BEAGLE [28] using all 1147 individual sequences from the 1000 bull’s genome project data [42] as the reference population. The 1000 bull’s data covered 24 purebred and 11 crossbred animals. The majority of the animals that were sequenced for the 1000 bull’s genome data are Holsteins (n = 389) and an average genome coverage of 11.0 was available for the entire dataset. A single-SNP regression was then applied to GWAS for this imputed region, using the same model as described above.

## Results

### Single SNP regression approach

**Fig. 1:**
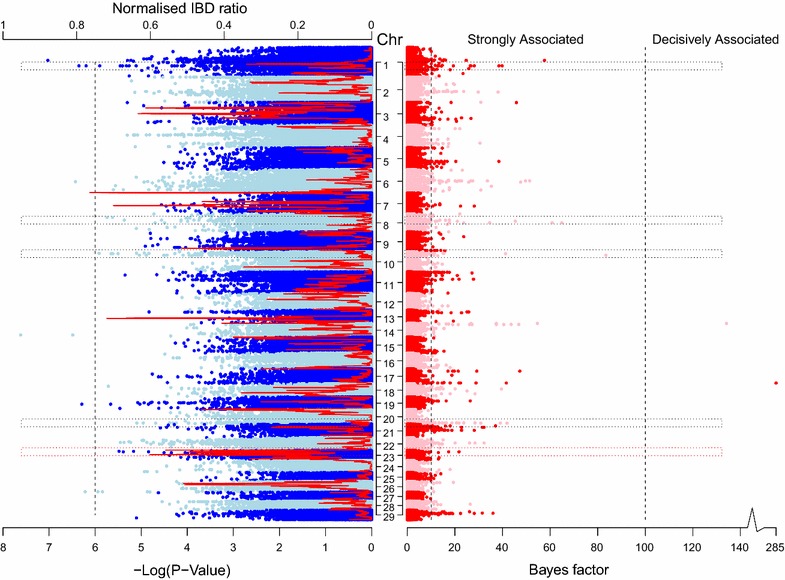
Manhattan plot of associations between each SNP and bTB susceptibility identified by three GWAS approaches. Single-SNP regression analysis (*left*) versus Manhattan plot of associations between each SNP and bTB susceptibility identified by the Bayesian approach (*right*). Proportion of shared IBD across the genome is represented by the *red line* within the single-SNP regression Manhattan plot. Only QTL that were significant in both the Bayesian and single-SNP regression approaches are highlighted by *black rectangles*. The only QTL that was found to be significant in the three approaches is highlighted by a *red rectangle*

**Fig. 2:**
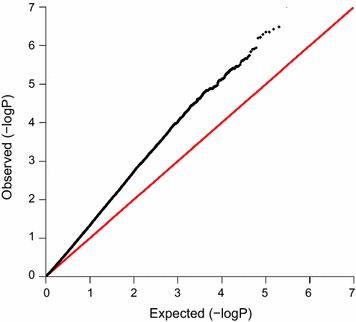
QQ-plot for P values (−Log_10_) estimated by the single-SNP regression analysis

Associations between each SNP and bTB susceptibility based on the single-SNP regression approach are illustrated in Fig. 1. All SNP effects estimated from the single-SNP regression approach are in Additional file 1: Table S1. Seventy-four SNPs were significant at the genome-wide level (P < 1 × 10^−6^). A QQ-plot of the observed against expected SNP P values is in Fig. 2. Twenty-eight QTL regions were identified based on SNPs with a genome-wide significance level of P < 1 × 10^−6^ with lengths ranging from 0.6 to 183 kb. The most significant (P < 1 × 10^−8^) SNP, BovineHD1400020824, was located on BTA14 and accounted for 0.089 % of the genetic variance; no QTL region could be identified around this SNP since it was not in strong LD with flanking SNPs. SNP BovineHD1400020824 was not located within a gene and the closest gene (*RUNX1T1*) was about 1 Mb away. The second most significant (P < 1 × 10^−8^) SNP, BovineHD0100019801, was located on BTA1 and accounted for 0.092 % of the genetic variance for bTB susceptibility. SNP BovineHD0100019801 was located within a 12-kb QTL region and within intron 11 of the *KALRN* gene. Its major allele A was associated with increased resistance to bTB infection, and homozygous animals for this allele had a mean EBV of −0.099 (n = 378, SD = 0.1390), whereas homozygous animals for the minor allele (MAF = 0.33) had a mean EBV of −0.0120 (n = 105, SD = 0.2010).

In the single-SNP regression analysis, we identified four genes (Table 1) that overlapped with or were within QTL regions. Of these genes, only two (*AP3B1* on BTA10 and *FKBP5* on BTA23), have a known role in immune response. The major T allele at the SNP Hapmap50596-BTA-121389 (P value = 4.09 × 10^−6^) that is located within the region on BTA23 was associated with increased resistance to bTB. Homozygous animals for the major allele of this SNP were on average more resistant to bTB infection with a mean EBV of −0.0909 (n = 703, SD = 0.1548) whereas homozygous animals for the minor allele (MAF = 0.1361) had a mean EBV of 0.0778 (n = 14, SD = 0.1924).

**Table 1:** Genes identified, chromosome, name of significant SNPs within QTL regions, P value, minor allele frequency (MAF), and size of QTL in bp

Chr^a^	Gene name	SNP name	P value (FDR = 1 %)	MAF	QTL size (bp)
1	*KALRN*	BovineHD0100019801	9.44 × 10^−8^	0.33	12,165
4	*DPP6*	BovineHD0400033997	7.42 × 10^−6^	0.45	32,568
		BovineHD0400033998	7.42 × 10^−6^	0.45	
10	*AP3B1*	BovineHD1000002985	2.23 × 10^−6^	0.24	13,726
		BovineHD1000002982	2.43 × 10^−6^	0.24	
		BovineHD1000030516	3.10 × 10^−6^	0.25	
23	*FKBP5*	Hapmap50596-BTA-121389	4.09 × 10^−6^	0.13	74,693

QTL regions identified in the single-SNP regression analysis applied to GWAS; the top 100 SNPs identified as significant are in Additional file 1: Table S1

^a^Chromosome number

Network analysis based on the results from the single-SNP regression analysis provided a list of networks that contained 292 genes, with “Antigen processing and presentation” (*PID Biocarta 4144*) as the most significantly overrepresented pathway (P < 1 × 10^−4^). The 10 most significantly represented pathways from the single-SNP regression analysis are in Table 2.

**Table 2:** Top ten biological pathways associated with bTB susceptibility based on the results of the single-SNP regression analysis applied to GWAS

Pathway name	Corrected P value	Source	Genes
Antigen processing and presentation	0.0011	PID BIOCARTA	*BT.91437*, *PSMB8*, *PSMB9*, *TAP1*
Arf1 pathway	0.0011	PID NCI	*AP2A1*, *AP2M1*, *CD4*, *CYTH2*, *KDELR1*
Validated targets of C-MYC transcriptional activation	0.0115	PID NCI	*BAX*, *BT.19231*, *EIF4A1*, *NME2*, *PDCD10*, *RUVBL2*, *TP53*
Nef Mediated CD4 Down-regulation	0.0145	REACTOME	*AP2A1*, *AP2M1*, *CD4*
ABC transporters	0.0285	KEGG	*ABCC3*, *BT.104466*, *BT.87418*, *BT.91437*, *TAP1*
Classical Kir channels	0.0321	REACTOME	*BT.62324*, *KCNJ12*
Antigen processing and presentation	0.0719	KEGG	*BOLA*-*DMA*, *BT.91437*, *CD4*, *TAP1*
ER-Phagosome pathway	0.0719	REACTOME	*BT.91437*, *PSMB8*, *PSMB9*, *PSMD2*, *TAP1*
Nef Mediated CD8 Down-regulation	0.0786	REACTOME	*AP2A1*, *AP2M1*
Tnf/stress related signalling	0.0838	PID BIOCARTA	*ATF1*, *MAP2K3*, *TANK*

### Bayesian approach

Bayes factors for each SNP are in Fig. 1 and all SNP effects estimated from the Bayesian approach applied to GWAS data are in Additional file 2: Table S2. The proportion of additive genetic variance accounted for by all SNPs using the Bayesian approach was equal to 15 % and 484 SNPs were identified as “strongly associated” (i.e., each SNP entered the Bayesian model in at least 0.01 of the Gibbs chains and therefore had a Bayes factor greater than 10). Two SNPs were “decisively associated” (i.e., each SNP entered the Bayesian model in at least 0.091 and therefore had a Bayes factor higher than 100). Fifty-seven QTL regions were identified around SNPs with a Bayes factor higher than 20 and ranged in length from 0.8 to 273 kb.

SNP BovineHD1700021001 that had the highest Bayes factor (i.e., 285) was located on BTA17 and accounted for 0.0018 % of the additive genetic variance. This SNP was located within a 3-kb QTL region and within intron 3 of the *RNF185* gene. Homozygous animals for the major allele of BovineHD1700021001 SNP were, on average, less resistant to bTB infection with a mean EBV of 0.0978 (n = 628, SD = 0.14901), and homozygous animals for the minor allele (MAF = 0.3234) had a mean EBV of −0.0191 (n = 16, SD = 0.1143).

We identified 40 genes that overlapped with or were within the 57 QTL regions detected (Table 3). Six of these genes were predicted to be involved in host immune response to infection: *PTN* on BTA4, *AP3B1* on BTA10, *HSF1*on BTA14, *SHARPIN* on BTA14, *TRAF4* on BTA20 and *FKBP5* on BTA23.

**Table 3:** Genes identified, within QTL identified using the Bayesian approach applied to GWAS

Chr^a^	Gene name	SNP name	Bayes factor	MAF	QTL length
1	*KALRN*	BovineHD0100019801	57.58	0.33	12,165
1	*TMPRSS2*	BovineHD0100041198	23.41	0.12	3575
2	*TMEFF2*	BovineHD0200023336	29.83	0.43	22,453
3	*NFIA*	BovineHD0300025132	26.77	0.41	17,003
		BovineHD0300024222	22.78	0.39	
3	*TMEM125*	ARS-BFGL-NGS-114804	20.69	0.39	28,366
4	*PTN*	BovineHD4100003190	30.57	0.41	6142
5	*DENND5B*	BovineHD0500022394	38.49	0.49	30,818
		BovineHD0500022395	20.38	0.49	
6	*PGM2*	BovineHD0600016172	51.47	0.42	62,372
		BovineHD0600016181	49.70	0.43	
6	*LIMCH1*	BovineHD0600017122	20.69	0.21	31,027
6	*SLC4A4*	BovineHD0600024174	35.37	0.34	6800
8	*PGM5*	BovineHD0800013359	21.01	0.47	16,615
10	*AP3B1*	BovineHD1000002985	41.29	0.24	13,726
14	*CPSF1*, *ADCK5*, *SLC52A2*	BovineHD1400000249	134.16	0.48	266,275
	*SCRT1*, *DGAT1*, *HSF1*,	BovineHD1400000246	37.41	0.48	
	*MROH1*, *MIR1839*, *MAF1*	BovineHD1400000246	37.41	0.48	
	*SHARPIN*, *CYC1*, *GPAA1*,				
	*EXOSC4*, *OPLAH*				
17	*OSBP2*	BovineHD1700020903	41.62	0.26	22,175
		BovineHD1700020906	28.88	0.26	
		BovineHD1700020907	28.88	0.26	
		BovineHD1700020908	22.36	0.26	
17	*RNF185*	BovineHD1700021001	285.55	0.32	3141
18	*SLC6A2*	ARS-BFGL-NGS-2712	20.07	0.096	12,244
18	*KCNK6*	BovineHD1800014284	28.46	0.26	101,126
20	*SDF2*, *SUPT6H*, *PROCA1*,	BovineHD2000019220	28.46	0.41	36,609
	*RAB34*, *RPL23A*, *TLCD1*,	BovineHD2000019231	23.72	0.41	
	*NEK8*, *TRAF4*	BovineHD2000019229	20.69	0.41	
23	*FKBP5*	Hapmap50596-BTA-121389	21.74	0.13	74,693
29	*ME3*	BovineHD2900002648	26.14	0.45	1263

Chromosome, name of SNPs with a significant Bayes factor within QTL regions, Bayes factor, minor allele frequency (MAF) and QTL size

^a^Chromosome number

Genes within 242 QTL defined from SNPs with a Bayes factor higher than 10 were considered for pathway analysis, which returned more than 350 statistically (P < 1 × 10^−4^) overrepresented pathways. Several pathways associated with immunity were identified, including *TNF alpha* (*Netpath 15913*, P < 10^−7^), adipocytokine signalling pathway (*KEGG 590*, P value <10^−6^), and *Fas* signalling pathway (*INOH 16231*, P value <10^−6^). The ten most significant pathways from the Bayesian approach are in Table 4.

**Table 4:** Top ten biological pathways associated with bTB susceptibility using results from the Bayesian approach applied to GWAS

Pathway name	Corrected P value	Source	Genes
Signalling events mediated by HDAC Class II	2.66 × 10^−14^	PID NCI	*BCL6*, *BCOR*, *GNB1*, *GNG2*, *HDAC4*, *HDAC7*, *HSP90AA1*, *NCOR2*, *RAN*, *YWHAB*, *YWHAE*
Cell cycle	4.63 × 10^−9^	KEGG	*EP300*, *GADD45A*, *GADD45B*, *GADD45G*, *HDAC2*, *MDM2*, *SFN*, *YWHAB*, *YWHAE*, *YWHAG*, *YWHAQ*, *YWHAZ*
Insulin receptor signalling	9.85 × 10^−9^	INOH	*PPP2R1A*, *PPP2R1B*, *PRKAA1*, *PRKAA2*, *SFN*, *YWHAB*, *YWHAE*, *YWHAG*, *YWHAQ*, *YWHAZ*
Glucocorticoid receptor regulatory network	1.05 × 10^−8^	PID NCI	*BT.26220*, *BT.55996*, *BT.60919*, *EGR1*, *HDAC2*, *HSP90AA1*, *MDM2*, *NCOA2*, *SFN*
Mechanism of gene regulation by peroxisome proliferators via ppara	1.18 × 10^−8^	PID BIOCARTA	*BT.26220*, *BT.55996*, *BT.78456*, *EP300*, *HSP90AA1*, *NCOR1*, *NCOR2*, *NR0B2*
Androgen receptor	2.88 × 10^−8^	NETPATH	*BT.55996*, *BT.61976*, *EP300*, *HDAC7*, *HSP90AA1*, *MDM2*, *NCOA2*, *NCOR1*, *NCOR2*, *NR0B2*, *RAN*, *RANBP9*
Leptin	3.72 × 10^−8^	NETPATH	*BT.26220*, *BT.55996*, *BT.60919*, *BT.61976*, *EP300*, *IKBKG*, *ITGB5*, *LIMK1*, *PRKAA1*, *PRKAA2*
Notch	4.10 × 10^−8^	NETPATH	*APP*, *BT.26220*, *BT.60919*, *BT.61976*, *EP300*, *HDAC2*, *NCOR1*, *NCOR2*, *SPEN*
Pathways in cancer	4.36 × 10^−8^	KEGG	*BT.25770*, *BT.26220*, *BT.56113*, *BT.60919*, *BT.78456*, *EP300*, *HDAC2*, *HSP90AA1*, *IKBKG*, *MDM2*, *PPARD*, *RAC2*, *RHOA*, *RXRB*, *RXRG*
TNF alpha	7.31 × 10^−8^	NETPATH	*BT.26220*, *BT.60919*, *BT.61976*, *BT.78456*, *EP300*, *GLUL*, *HDAC2*, *HSP90AA1*, *IKBKG*, *YWHAB*, *YWHAE*, *YWHAG*, *YWHAQ*, *YWHAZ*

### IBD analysis

Eighteen prolific sires in the test group (i.e. sires with more than 50 daughters in more than 10 herds) were previously identified by Richardson et al. [5] as belonging to a small group of high bTB individuals with an extremely high mean daughter prevalence of bTB infection (≥60 %, compared to an average of 19 %). Eleven of these outlier high bBT sires were strongly related and fell into four lineages. One lineage traced six of these sires back to one ancestor and the four lineages had multiple shared sires. We hypothesised that the high level of shared susceptibility among these lineages could be due to specific genomic regions that are shared by common descent from founders. The normalized proportion of IBD segments shared across the genome is plotted in Fig. 1. The most represented, i.e. the top 20 shared IBD blocks, are in Table 5 with a 5-Mb block on BTA13 being the most represented. Fifty-four percent of the high bTB sires used in the IBD analysis shared this haplotype compared to 12 % of sires with a low (<30 %) prevalence of bTB infection in their daughters.

**Table 5:** Top 20 IBD haplotype blocks shared between sires with a high daughter prevalence of bTB infection

Chr^a^	Start SNP	End SNP	Start position	End position	High prop	Low prop
13	BovineHD1300014366	BovineHD1300014692	49589499	51444238	0.54	0.12
13	BovineHD1300013610	BovineHD1300014364	46502709	49576039	0.52	0.12
7	BovineHD0700014051	BovineHD0700014305	48615285	49344575	0.52	0.15
7	BovineHD0700014306	BovineHD0700015867	49347339	54833963	0.50	0.18
13	BovineHD1300013374	BovineHD1300013492	45829062	46168983	0.47	0.14
7	BovineHD0700019982	BovineHD0700020720	68220084	70459483	0.45	0.10
7	BovineHD0700013664	BovineHD0700014049	47130142	48606465	0.43	0.14
3	BovineHD4100002136	BovineHD0300018020	56353864	60108292	0.41	0.09
9	BovineHD0900024988	BovineHD4100007649	88657158	90474884	0.41	0.10
13	BovineHD1300014695	BovineHD1300016474	51460099	57393129	0.41	0.11
7	BovineHD0700019273	BovineHD0700019979	66064958	68207807	0.41	0.09
7	BovineHD0700015911	BovineHD0700016486	55087353	57559898	0.40	0.17
7	ARS-BFGL-NGS-33898	BovineHD0700033819	65520210	66054838	0.40	0.09
7	BovineHD0700020722	BovineHD0700021987	70463539	74679215	0.38	0.09
3	BovineHD0300018022	BovineHD0300018594	60114338	61974915	0.38	0.09
7	BovineHD4100006127	BovineHD0700019111	63918463	65517703	0.38	0.09
3	BovineHD0300024252	BovineHD0300024614	84878201	86233867	0.38	0.17
13	BovineHD1300011097	BovineHD1300012983	38486971	44519866	0.38	0.13
23	BovineHD2300001843	BovineHD2300002752	7308515	11189811	0.38	0.09

The percentages of each IBD block in the high bTB and low bTB groups are represented by the high prop and low prop groups, respectively

^a^Chromosome

### Cross-method comparisons

The Bayesian and single-SNP regression approaches that were applied to GWAS data identified 17 SNPs that were significant (i.e. Bayes factor >10 and P value <1 × 10^−5^) with both approaches (Table 6). Three QTL on BTA1, 10 and 23 were found with both the Bayesian and single-SNP regression approaches. One IBD block (~3 Mb long) on BTA23 that was shared by 38 % of the high bTB sires overlapped with QTL regions detected in the other analyses and contained 63 genes, five of which were predicted to be involved in host immune response (*TAPBP*, *DEF6*, *FKBP5*, *MAPK14* and *MAPK13*).

**Table 6:** SNPs significantly associated with bTB susceptibility detected with both the Bayesian and single-SNP regression approaches

SNP	Chr^a^	Genetic position	Bayes factor	P value^b^
BovineHD0100019699	1	69368803	24.77	6.03 × 10^−6^
BovineHD0100019801	1	69548446	57.58	9.55 × 10^−8^
BovineHD0100029053^c^	1	101890992	38.49	1.26 × 10^−6^
Hapmap53234-rs29020933^c^	1	101892420	26.45	7.94 × 10^−6^
BovineHD0100029057^c^	1	101896647	28.14	5.50 × 10^−6^
BovineHD0100029059^c^	1	101899126	39.89	4.47 × 10^−7^
BovineHD010002906^c^	1	101899991	26.77	6.46 × 10^−7^
BovineHD0100029061^c^	1	101903117	19.34	4.47 × 10^−7^
BovineHD0100029062^c^	1	101910522	12.74	3.55 × 10^−6^
Hapmap43413-BTA-95698^c^	1	101918115	19.34	3.55 × 10^−6^
BovineHD0100029132	1	102058031	13.05	3.98 × 10^−6^
BovineHD0300000514	3	1943906	45.86	5.01 × 10^−6^
BovineHD0800010906	8	36652588	17.69	4.37 × 10^−6^
BovineHD1000002985^c^	10	9147391	41.29	2.24 × 10^−6^
BovineHD2000019456	20	67224771	21.74	8.13 × 10^−6^
Hapmap50596-BTA-121389^c^	23	9591806	21.74	4.17 × 10^−6^
BovineHD2600014591	26	50406685	11.11	1.45 × 10^−6^

^a^Chromosome

^b^FDR level of 1 %

^c^Indicates a QTL that could be identified based on this SNP

### Imputation of the candidate region on BTA23

**Fig. 3:**
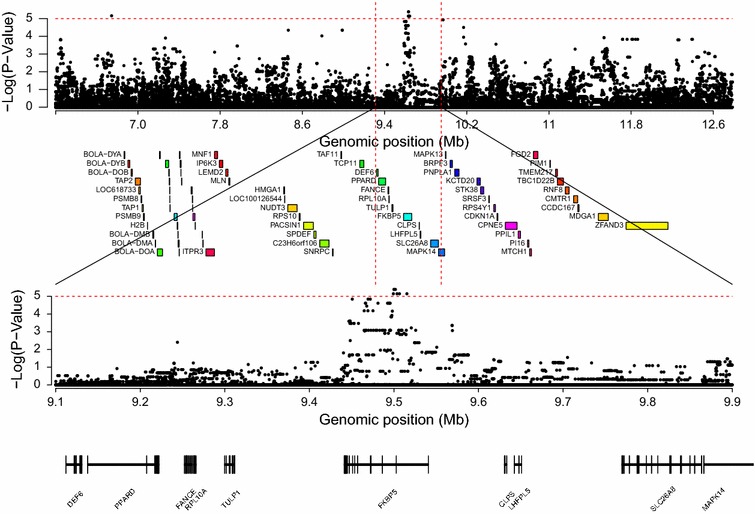
Manhattan plot of associations between SNPs and bTB susceptibility within regions on BTA23. Manhattan plot of associations between each SNP and bTB susceptibility identified within a 5 Mb (*top*) and 1 Mb (*bottom*) region on BTA23 imputed to full sequence using the single-SNP regression analysis with annotated genes indicated under the plot. Genome-wide significance level is indicated by the *red dashed line*

The candidate QTL region on BTA23 was the only region that was significantly associated with bTB susceptibility in the three approaches used and this was selected for targeted imputation. Strengths of association between bTB susceptibility and each SNP within the 3-Mb regions that flanked each side of the QTL on BTA23 and were imputed to full sequence are plotted in Fig. 3. Twenty-two SNPs in the imputed BTA23 region had a significance level P lower than 1 × 10^−5^. All but two of the top ten of the most significant SNPs (P < 1 × 10^−5^) were located within introns 1, 2, 5 or 8 of the *FKBP5* gene (including the two most significant SNPs (P < 4 × 10^−6^) located at positions 9590819 and 9591806) and the other two SNPs were located within intron 8 of the *MAPK14* gene. Furthermore, a distinct peak of SNPs with strong P values was detected near the significant SNPs located in the *FKBP5* gene (Fig. 3).

## Discussion

In developed countries, current strategies for reducing bTB infection in cattle primarily focus on the detection of infection in herds (through annual herd tests and abattoir surveillance), to effectively control infection in known infected herds, and to limit the spread of infection between herds and regions. These strategies are increasingly associated with active programmes to manage infection in known wildlife reservoirs. Other strategies are currently being investigated including increased host resistance through focused breeding practices [5] or the use of vaccination either in cattle or wildlife reservoirs [43]. Because both across- and within-breed differences in susceptibility to bTB exist, breed substitution or within-breed selection are viable options to supplement on-going bTB eradication programs. Moreover, there is considerable heritable genetic variation in the susceptibility to bTB, which suggests that variation at the genomic level governs these differences in bTB susceptibility among animals. However, only three GWAS in cattle [16, 17, 19] have attempted to locate genomic regions that contribute to the additive genetic variance of susceptibility to bTB. Elucidating this genomic variation may help to develop new pharmaceuticals, and incorporation of genomic information into genetic evaluations may result in greater rates of genetic gain.

### Methodological comparisons

In a single-SNP regression analysis, each SNP is included and tested for significance individually in the model. In the Bayesian approach, all SNPs are fitted simultaneously within the same model. Among the significant SNPs that were identified in the single-SNP regression and Bayesian approaches, 17 common SNPs were found (Table 6). However, the most significant SNP detected in the single-SNP regression analysis was not the most significant SNP in the Bayesian approach and vice versa. Significant associations that are identified by using single-SNP regression or other frequentist approaches generally only explain a small fraction of the genetic variation of the quantitative trait [44]; in contrast, Bayesian approaches applied to GWAS can account for most of the genetic variation by simultaneously fitting all the SNPs as random effects.

### Comparison of our results with those of previous GWAS

Results from the current study do not corroborate those of Finlay et al. [16], who identified a region on BTA22 for bTB susceptibility. We consider that our results supersede this prior work because they have greater statistical power due to a larger number of genotypes (n = 841 vs. n = 307), greater genome coverage, and larger amount of underlying epidemiological data. Based on field case/control data (592 cases and 559 controls), whereas we used SICTT results as quantitative trait, Bermingham et al. [17] suggested two novel resistance loci for bTB infection with chromosome-wide significance, located on BTA2 and 13, which we did not replicate here. This may be due to differences in the phenotypes used, differences in population structure, or limited analytical power. Although the number of test animals included were similar, our study is based on the phenotypes of several thousand animals (n = 105,526). Kassahun et al. [19] reported an admixture mapping analysis for bTB susceptibility in Ethiopian Zebu/Holstein–Friesian hybrids that suggested a *toll*-*like receptor* gene cluster on BTA6, which we did not replicate either here. Finally, the genomic regions that we found to be associated with bTB susceptibility differed from those documented to be associated with paratuberculosis in cattle [13–15], which shares some similarities with bTB.

### Novel candidate quantitative trait loci

The Bayesian, single-SNP regression and IBD approaches identified a region on BTA23 that is significantly associated with bTB susceptibility in dairy cattle and contains two genes *FKBP5* and *MAPK14*. The *FKBP5* gene encodes a protein from the immunophillin protein family, a family of proteins that is often targeted by immunosuppressant drugs. FKBP5 is a *cis*–*trans* prolyl isomerase that binds to the immunosuppressants *FK506* and rapamycin [45]. *FKBP5* is also involved in the *TNF alpha/NF*-*kB* signalling pathway, which is a major pathway involved in host immune response to disease and other harmful stressors, and is highly expressed in T-lymphocytes [45]. All the significant SNPs that were detected in the analysis based on the regions that flanked the *FKBP5* gene and were imputed to full sequence were located in introns, which is consistent with previously described associations between introns and disease traits [46–48]. Furthermore, the distinct peak of SNPs with strong P values that was detected near the significant SNPs located within *FKBP5* (Fig. 3), increases the likelihood that this gene is truly associated with host susceptibility to bTB.

However, it must be noted that although the QTL region that contains the *FKBP5* gene was the only region identified in all three analyses, it was not the most significant in any individual analysis. The most significant SNPs (P < 1 × 10^−8^; n = 2) that were detected by the single-SNP regression analysis were either located within genes that were not directly involved in immune response (BTA1; BovineHD0100019801, located within the gene *KALRN*), or in QTL regions that do not contain genes (BTA14; BovineHD1400020824). The most significant SNP found in the Bayesian analysis (BTA17; BovineHD1700021001, Bayes factor = 285) was identified within the *RNF185*gene that has no known role in immune response. However, because this SNP is strongly associated with bTB susceptibility, this region should be further investigated and might provide more insight into the host genetic susceptibility to bTB. Moreover, while the QTL region that harbored the second most significant SNP in the Bayesian analysis (BovineHD1400000249, Bayes factor = 134) contained two genes (*HSF1* and *SHARPIN*) that are involved in immune response, it is also associated with the *DGAT1* gene which is known to play a key role in milk fat production [49].

A QTL region on BTA1 that was estimated as significant (P < 1 × 10^−5^, Bayes factor >10) in both the Bayesian and single-SNP regression approaches contained nine of the 17 significant SNPs common to both approaches, but it was not detected in the IBD approach. Although, to date, no relevant genes were identified within this QTL, because of this large number of significant SNPs that are shared between the Bayesian and single-SNP regression approaches, it is an interesting candidate for further investigation.

### Network analysis

Identification of TB susceptibility genes in humans has been documented to be more difficult compared to that for other infections, even with large sample sizes [50]. This is possibly the result of selection against variants with large or even moderate effects during this long-standing infection. However, the most significantly associated pathway based on our results from the single-SNP regression analysis was the antigen processing and presentation pathway. Multiple mutations in the genes involved in this pathway could lead to decreased expression or absence of expression of antigens at the surface of infected cells that allow cell recognition and degradation by the host immune system. *M. bovis* replicates within the lysosomes of the host macrophages, which suggests that an impaired antigen processing and presentation pathway could allow *M. bovis* to survive longer in the host, leading macrophages to be less readily degraded, thus increasing the host’s susceptibility to infection.

## Conclusions

Due to the likely polygenic nature of bTB susceptibility, several approaches applied to GWAS were conducted to identify novel associations that would otherwise not be identified by using a single-SNP approach. A candidate region on BTA23 was identified and imputation of this region to sequence data pinpointed the association signal in the introns of the *FKBP5* gene, which is involved in immune response. However, it must be noted that this association signal on BTA23 was not the most significant in any one of the single analyses and that the power of analysis was not yet sufficient to identify clear associations between SNPs and bTB susceptibility. Aside from this observation, our study constitutes another step towards the identification of the genetic mechanisms that underlie the elusive bTB susceptibility trait.

## Additional files

10.1186/s12711-016-0197-x All_SNP_effects_SSR.csv. A list of all the SNP effects estimated from single-SNP regression analysis. Columns are, chromosome, solution, error, t-like, P value, Log_10_ (P value), SNP id, genetic position, q-value.
10.1186/s12711-016-0197-x All_SNP_effects_Bayesian.csv. Description: A list of all the SNP effects estimated from the Bayesian approach applied to GWAS data. Columns are chromosome, SNP id, genetic position, SNP effect, effect variance, model frequency, window frequency, genetic frequency, genetic variance, effect delta, standard deviation of effect delta, t-like, shrink effect, Bayes factor.
